# Functional polymorphisms of ATP citrate lyase gene predicts clinical outcome of patients with advanced colorectal cancer

**DOI:** 10.1186/s12957-015-0440-x

**Published:** 2015-02-12

**Authors:** Shuang Xie, Feng Zhou, Jiaojiao Wang, Haiyan Cao, Yibing Chen, Xiaonan Liu, Zhaohui Zhang, Jingyao Dai, Xianli He

**Affiliations:** Department of General Surgery, Tangdu Hospital, Fourth Military Medical University, 169 West Changle Street, Xi’an, 710032 China; State Key Laboratory of Cancer Biology, Experimental Teaching Center of Basic Medicine, Fourth Military Medical University, Xi’an, 710032 China; Department of General Surgery, Huaihai Hospital, Xuzhou Medical College, Xuzhou, Jiangsu 221004 China; Xijing Hospital of Digestive Disease, Fourth Military Medical University, Xi’an, 710032 China; Department of Hepatobiliary Surgery, Xijing Hospital, Fourth Military Medical University, Xi’an, 710032 China

**Keywords:** Single nucleotide polymorphism, *De novo* lipogenesis, Colorectal cancer, ACLY, Prognosis

## Abstract

**Background:**

Previous studies have demonstrated that ATP citrate lyase (ACLY) plays an important role in the development of many cancers. Our current study aims to assess the effects of functional single nucleotide polymorphisms (SNPs) in *ACLY* gene on recurrence and survival of colorectal cancer (CRC) patients.

**Methods:**

A total of 697 resected Chinese CRC patients were included in this study. Two functional single nucleotide polymorphisms in *ACLY* gene were examined using the Sequenom iPLEX genotyping system. Multivariate Cox proportional hazards model and Kaplan-Meier curve were used for the prognosis analysis.

**Results:**

Multivariate Cox regression analysis showed that there was no significant association between SNPs in *ACLY* gene and the prognosis of total patient cohort. However, in patients with stage III + IV diseases, the two functional SNPs (rs2304497 and rs9912300) exhibited a significant association with the risks of death (HR = 0.47, 95% CI = 0.24–0.90 and HR = 0.59, 95% CI = 0.37–0.92, respectively) and recurrence (HR = 0.46, 95% CI = 0.24–0.86 and HR = 0.54, CI = 0.35–0.83, respectively). Kaplan-Meier analysis indicated that those CRC patients carrying heterozygous (WV) or homozygous variant (VV) genotypes in rs2304497 and rs9912300 had significantly better overall survival (OS) and recurrence-free survival (RFS). Moreover, we observed remarkable cumulative effects of these two SNPs on overall survival and recurrence-free survival (*P* for trend = 0.012 and 0.003, respectively). Compared with patients carrying zero unfavorable genotype, those carrying two unfavorable genotypes had a 2.24-fold and 2.33-fold increase of death and recurrence risks, respectively.

**Conclusions:**

The SNPs in *ACLY* gene may serve as independent prognostic markers for patients with advanced stage CRC.

**Electronic supplementary material:**

The online version of this article (doi:10.1186/s12957-015-0440-x) contains supplementary material, which is available to authorized users.

## Background

Fatty acids play an important role in a variety of cellular processes. *De novo* lipogenesis (DNL) is an endogenous pathway whereby carbohydrates are converted to fatty acids [1]. DNL occurs at low rates in most non-dividing cells of normal tissues that primarily uptake lipids from circulation. In contrast, enhanced DNL is one of the most common properties of cancer cells. Many studies have demonstrated that cancer cells prefer DNL-derived fatty acids instead of extracellular lipid supply [2]. In these rapidly proliferating cells, citrate generated by the tricarboxylic acid cycle is preferentially exported from the mitochondria to the cytosol and then cleaved by ATP citrate lyase (ACLY) to produce cytosolic acetyl coenzyme A, which is the building block for DNL [3]. Therefore, ACLY couples energy metabolism with fatty acid synthesis and plays a critical role in supporting cell growth. Distinctive elevation of ACLY expression and activity has been reported in lung, ovarian, prostate, bladder, breast, liver, stomach, and colon cancers [4-10], and its inhibition by siRNAs or the selective inhibitor SB-204990 suppresses the growth and survival of tumor cells *in vitro* and *in vivo* [11]. Furthermore, Migita et al. have reported that the overexpression of ACLY is well-correlated with stage, differentiation grade, and a poorer prognosis in non-small cell lung cancer [4]. All these data strongly support the idea that the *ACLY* is involved in the development and progression of human cancers.

Colorectal cancer (CRC) is a common malignancy as well as a leading cause of cancer mortality in both America and China [12,13]. According to National Central Cancer Registry Database of China, CRC incidence has increased annually, and the uptrend in rural areas is more obvious than it in urban areas in China [14]. Fortunately, evidences have shown that the mortality rate of CRC has decreased in Asian countries, possibly due to the early screening and detection as well as the use of more advanced surgical and systemic modalities [15]. However, a considerable proportion of CRC patients develop recurrence or metastasis within 5 years after surgical treatment, highlighting the importance of the establishment of novel biomarkers to identify those patients who are most likely to develop recurrence or metastasis and thus should receive more aggressive therapies.

Single nucleotide polymorphism (SNP) is the most common genetic variation, and numerous previous studies have shown that SNPs may be used as surrogates of patients’ genetic background to predict therapeutic response and prognosis [16-18]. Our previous studies also found that some SNPs are significantly associated with overall survival in different kinds of cancer patients [19-21]. However, whether functional variations in *ACLY* gene have any influence on CRC clinical outcomes remains unclear. In this study, we assessed the effects of functional SNPs in *ACLY* gene on recurrence and survival in a cohort of 697 resected Chinese CRC patients.

## Methods

### Study population

Between May 2006 and June 2012, CRC patients were enrolled at Xijing and Tangdu Hospitals affiliated to The Fourth Military Medical University in Xi’an, China. The enrolled patients have to match the following criteria: 1) histologically confirmed with primary colorectal adenocarcinoma and no history of other cancers; 2) received curative surgical resection treatment but without any preoperative anticancer treatment; and 3) with complete clinical and follow-up data as well as common epidemiological data. All patients enrolled in this study underwent standardized oncologic resection at our department. None of them received emergency operations. The affected colonic segment was resected with an adequate safety margin in combination with complete, radical lymphadenectomy. Patients with a tumor located in the upper third of the rectum underwent rectum resection together with a partial mesorectal excision (PME); in patients with a tumor of the middle or lower third, a total mesorectal excision (TME) was performed. In this prognosis study, we excluded 16 patients who died within 1 month after surgery. Finally, 697 patients were included in the present study. Before surgical resection, 5 mL of peripheral blood sample was collected from each patient for DNA preparation. This study was approved by the Ethics Committee of the Fourth Military Medical University and the signed informed consent was obtained from each participant.

### Epidemiologic and clinical data collection

Demographic and personal data were collected through in-person interview using a standardized epidemiological questionnaire, including gender, sex, ethnicity, and residential region. Detailed clinical information was collected through medical chart review or consultation with treating physicians, including time of diagnosis, time of surgery and/or chemotherapies, time of recurrence and/or death, tumor stage, differentiation, location site, lymph node invasiveness, treatment protocol, and serum carcinoembryonic antigen (CEA). A standard follow-up was performed by a trained clinical specialist through on-site interview, direct calling, or medical chart review at 6-month intervals. The latest follow-up data in this analysis was obtained in January 2014.

### SNP selection and genotyping

Functional SNPs in *ACLY* gene was selected using a set of web-based SNP selection tools (http://snpinfo.niehs.nih.gov/snpfunc.htm) as described previously [22]. The 5′ and 3′ flanking regions were arbitrarily set at 2,000 bp for all genes. Only validated SNPs were selected, and SNPs with minor allele frequency (MAF) <5% in Chinese population were excluded. In the case of multiple potentially functional SNPs within the same haplotype block (defined by the linkage coefficient *r*^2^ > 0.8), only one SNP was included. Functional SNPs included missense SNPs in exons, SNPs in miRNA-binding sites of 3′UTR, SNPs in transcription factor binding site of 5′ flanking region, as well as SNPs in splice sites.

Through the selection process, two SNPs in *ACLY* gene were selected for further genotyping, including one missense SNP (rs2304497) and one SNP in transcription factor binding site (rs9912300). Genotyping was performed using genomic DNA on Sequenom iPLEX genotyping system (Sequenom, San Diego, CA, USA). The laboratory personnel conducting genotyping were blinded to patient information. Strict quality control measures were implemented during genotyping with more than 99.0% concordance with the main genotyping results.

### Statistical analysis

The clinical outcomes of CRC patients include two major endpoints, which are overall survival (OS) and recurrence-free survival (RFS). Overall survival time was defined as the time from initial surgery to death from any cause. Recurrence-free survival time was defined as the time from initial surgery to local recurrence, distant recurrent metastasis. All statistical analyses were performed using the SPSS Statistics 19.0 software (IBM, Armonk, NY, USA). The dominant genetic model was applied to assess the association of single SNPs with clinical outcome of CRC patients. Hazard ratios (HRs) and 95% confidence interval (95% CI) were estimated from a multivariate Cox proportional hazards model, adjusting for gender, age, hospital site, tumor position, TNM stage, tumor differentiation, and treatment after surgery where appropriate. The cumulative effect of unfavorable genotypes on OS or RFS was estimated in Cox model. Haplotypes were determined using the HaploView software package (version 4.2). Kaplan-Meier curve and log-rank test were used to assess the differences of overall survival. All *P* values in this study were two-sided. *P* ≤ 0.05 was considered the threshold of statistical significance.

## Results

### Distribution of patient characteristics and prognosis analysis

The distribution of demographic and clinical characteristics of patients was presented in Table 1. A total of 697 CRC patients were included in this study, with a median age of 60 years (ranging from 15 to 73 years). Among them, 383 patients (54.9%) were male, and 65.1% were from Tangdu Hospital. More than half (*n* = 378, 54.2%) had rectal cancer. There were 453 (65.0%) and 244 (35.0%) patients with stage I–II and stage III–IV diseases, respectively. Nearly 80% of patients (*n* = 555) had moderately and poorly differentiated tumors, and 528 patients (75.8%) received adjuvant chemotherapy with 5-fluorouracil (5-FU)-based regimen after surgery.

**Table 1:** **Distribution of patients’ characteristics and prognosis analysis**

**Parameter**	**All patients,** ***n*** **(%)** ***n*** **= 697**	**OS**				**RFS**			
		**Death,** ***n*** **(%)** ***n*** **= 205**	**HR** ^**a**^	**95% CI**	***P*** **value**	**Recurrence,** ***n*** **(%)** ***n*** **= 240**	**HR** ^**a**^	**95% CI**	***P*** **value**
Gender									
Female	314(45.1)	87(42.4)	Ref.			100(41.7)	Ref.		
Male	383(54.9)	118(57.6)	1.24	0.94–1.64	0.13	140(58.3)	1.29	1.00–1.67	0.06
Age									
<60	358(51.4)	99(48.3)	Ref.			123(51.3)	Ref.		
≥60	339(48.6)	106(51.7)	1.05	0.80–1.39	0.72	117(48.8)	0.91	0.70–1.17	0.45
Hospital site									
Tangdu	454(65.1)	126(61.5)	Ref.			140(58.3)	Ref.		
Xijing	243(34.9)	79(38.5)	1.01	0.75–1.36	0.93	100(41.7)	1.25	0.95–1.64	0.11
Tumor position									
Colon	319(45.8)	93(45.4)	Ref.			108(45.0)	Ref.		
Rectum	378(54.2)	112(54.6)	1.08	0.82–1.42	0.60	132(55.0)	1.05	0.81–1.36	0.70
TNM stage									
I + II	453(65.0)	96(46.8)	Ref.			120(50.0)	Ref.		
III + IV	244(35.0)	109(53.2)	*3.82*	*2.75–5.33*	*<0.001*	120(50.0)	*3.13*	*2.32–4.23*	*<0.001*
Tumor differentiation									
Well	142(20.4)	35(17.1)	Ref.			45(18.8)	Ref.		
Moderate/poor	555(79.6)	170(82.9)	1.38	0.94–2.03	0.10	195(81.3)	1.26	0.89–1.78	0.19
Treatment after surgery									
None	169(24.2)	59(28.8)	Ref.			64(26.7)	Ref.		
Chemotherapy	528(75.8)	146(71.2)	*0.36*	*0.25–0.52*	*<0.001*	176(73.3)	*0.45*	*0.32–0.63*	*<0.001*

Significant *P* values (<0.05) are in italics.

*Abbreviations*: *CI*, confidence interval; *HR*, hazard ratio; *OS*, overall survival; *RFS*, recurrence free survival; *Ref*., reference.

^a^Adjusted by gender, age, hospital site, tumor position, TNM stage, tumor differentiation, and treatment after surgery where appropriate.

During the median follow-up of 35.7 months (ranging from 3.1 to 83.4 months), 205 patients (29.4%) died and 240 patients (34.4%) developed recurrence. Furthermore, Cox regression analyses showed that TNM stage and treatment after surgery had significant influence on OS and RFS for CRC patients (*P* < 0.001 for both). Patients with advanced stage CRC had significantly shorter OS and RFS (both *P* < 0.001). The adjuvant chemotherapy had a significantly decreased risk of death (HR = 0.36; 95% CI = 0.25–0.52) and recurrence (HR = 0.45; 95% CI = 0.32–0.63).

### Association of single SNP with clinical outcomes

**Figure 1:**
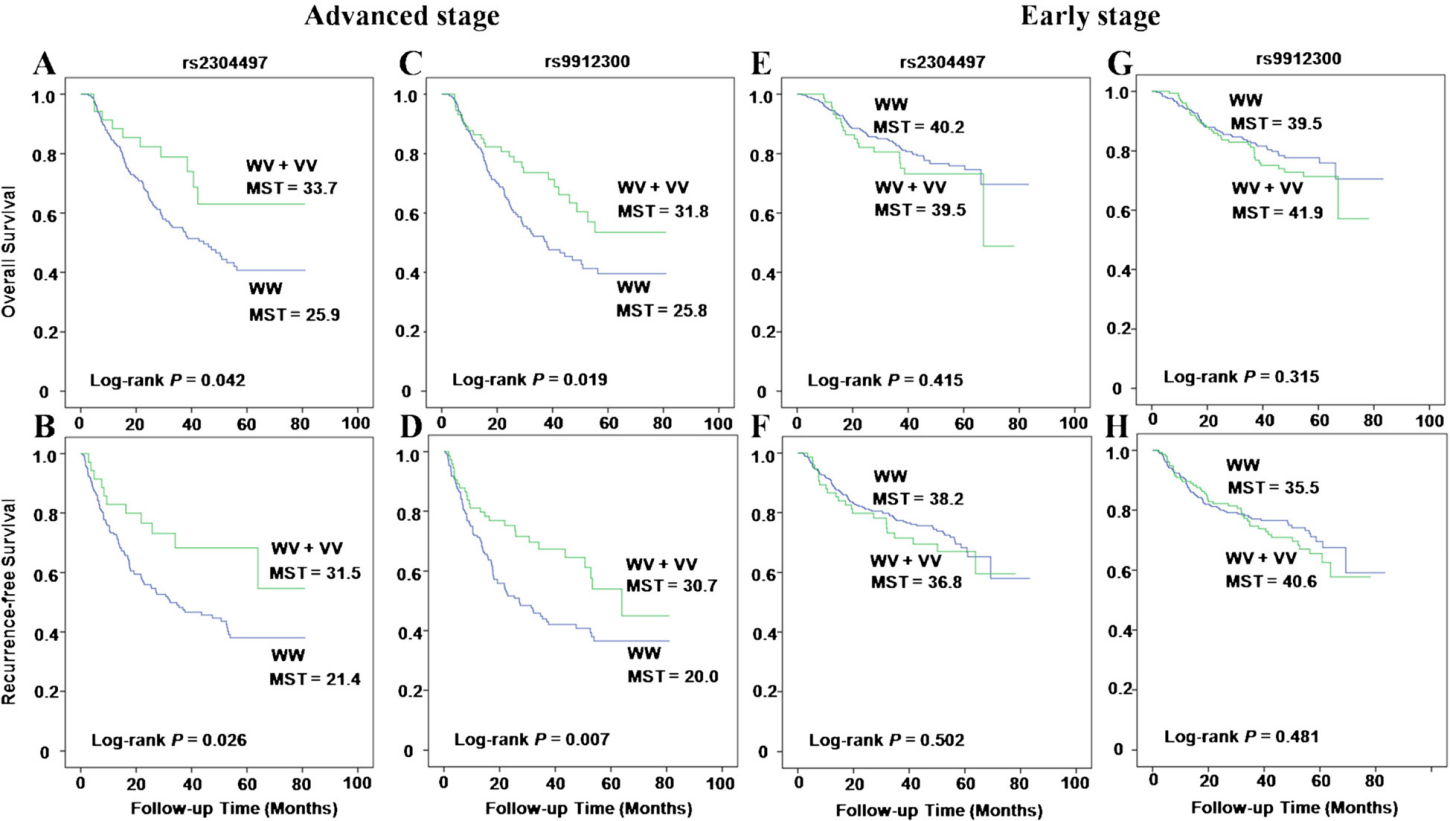
**Kaplan-Meier curves of overall and recurrence-free survival.** CRC patients with advanced stage diseases **(A–D)** and early stage diseases **(E–H)**.

We assessed the effect of two SNPs in *ACLY* gene on the death and recurrence in CRC patients using the multivariate Cox regression model (Table 2). After adjusting for gender, age, hospital site, tumor position, TNM stage, tumor differentiation, and treatment after surgery, no significant association was observed between SNPs and CRC patient outcomes. To further evaluate the modifying effect of host characteristics on association of SNPs in *ACLY* gene with the prognosis, we performed a stratified analysis in patients with early stage and advanced stage tumor. We found that in patients with advanced stage tumor (stage III + IV), the heterozygous variant (WV) and homozygous variant (VV) genotypes in both rs2304497 and rs9912300 reduced the death risk of CRC in the dominant model (HR = 0.47, 95% CI = 0.24–0.90 and HR = 0.59, 95% CI = 0.37–0.92, respectively). They also exhibited significant associations with recurrence risk (HR = 0.46; 95% CI = 0.24–0.86 and HR = 0.54; CI = 0.35–0.83, respectively). Kaplan-Meier analysis showed similar results, indicating that CRC patients with advanced stage carrying heterozygous (WV) or homozygous variant (VV) genotypes in rs2304497 and rs9912300 had significantly better OS and RFS than those with corresponding homozygous wild-type (WW) genotype (Figure 1A–D).

**Table 2:** **Associations between ACLY gene genotypes and clinical outcomes of CRC patients**

**Patients/SNPs**	**Genotypes**	**OS**			**RFS**		
		**Death/all**	**HR** ^**a**^ **(95% CI)**	***P*** **value**	**Recurrence/all**	**HR** ^**a**^ **(95% CI)**	***P*** **value**
In all patients							
rs2304497	WW	173/578	Ref.		203/578	Ref.	
	WV + VV	29/111	0.87(0.59–1.29)	0.49	34/111	0.85(0.59–1.23)	0.39
rs9912300	WW	140/461	Ref.		165/461	Ref.	
	WV + VV	63/229	0.87(0.64–1.17)	0.35	73/229	0.81(0.61–1.07)	0.13
In patients with early stage (I + II)							
rs2304497	WW	74/370	Ref.		94/370	Ref.	
	WV + VV	19/76	1.32(0.79–2.19)	0.29	23/76	1.20(0.75–1.90)	0.45
rs9912300	WW	56/292	Ref.		72/292	Ref.	
	WV + VV	38/155	1.28(0.84–1.94)	0.25	46/155	1.15(0.79–1.67)	0.47
In patients with advanced stage (III + IV)							
rs2304497	WW	99/208	Ref.		109/208	Ref.	
	WV + VV	10/35	*0.47(0.24–0.90)*	*0.02*	11/35	*0.46(0.24–0.86)*	*0.02*
rs9912300	WW	84/169	Ref.		93/169	Ref.	
	WV + VV	25/74	*0.59(0.37–0.92)*	*0.02*	27/74	*0.54(0.35–0.83)*	*0.01*

Significant *P* values (<0.05) are in italics.

*Abbreviations*: *CI*, confidence interval; *HR*, hazard ratio; *OS*, overall survival; *RFS*, recurrence free survival; *Ref*., reference.

^a^Adjusted by gender, age, hospital site, tumor position, TNM stage, tumor differentiation, and treatment after surgery where appropriate.

### Cumulative effect of unfavorable genotypes on overall survival and recurrence-free survival in patients with advanced diseases

**Figure 2:**
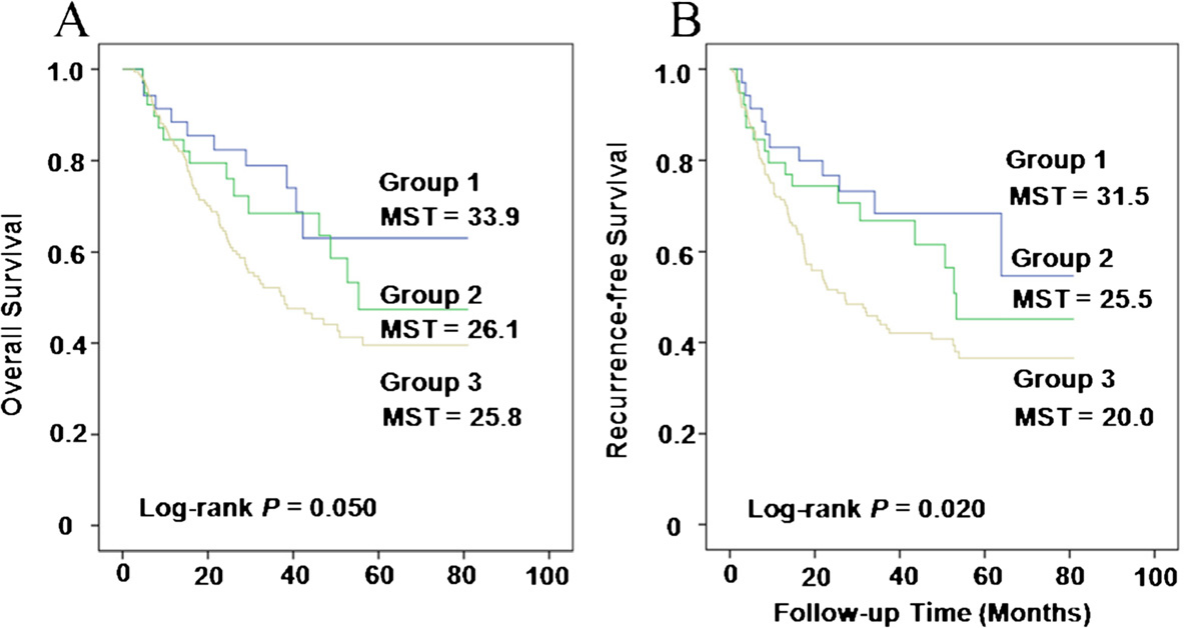
**Cumulative effect of unfavorable genotypes of** ***ACLY*** **gene.** Effect on overall **(A)** and recurrence-free **(B)** survival of survival in CRC patients with advanced diseases analyzed by Kaplan–Meier curves.

To further assess the cumulative effects of genetic variants on CRC overall survival in patients with advanced diseases, we did a joint analysis by including the two SNPs showing a significant association in single-SNP analysis (Table 2). The unfavorable genotypes were defined as the homozygous wild-type (WW) for both rs2304497 and rs9912300. When using group 1 (with zero unfavorable genotype) as reference, CRC patients in group 3 (with two unfavorable genotype) had a 2.24-fold increase of death (95% CI = 1.15–4.36; *P* = 0.17). A significant dose–response trend was observed (*P* for trend = 0.012) (Table 3). Furthermore, the risk of recurrence increased with the increasing number of unfavorable genotype (*P* for trend = 0.003). Kaplan–Meier analysis showed that there was a significantly decreased OS and RFS in patients carrying two unfavorable genotypes, compared with those carrying zero unfavorable genotype (log-rank *P* = 0.05 and *P* = 0.02, respectively, Figure 2A, B). We performed a stratified analysis to assess the effects of genetic variants on OS and RFS in patients with/without chemotherapy (Additional file 1: Table S1). No statistical significance was observed for SNP rs2304497 and rs9912300 in both subgroups. These results suggested that there might be no modifying effect of chemotherapy on the prognostic significance of both SNPs.

**Table 3:** **Cumulative effect of unfavorable genotypes on overall survival of CRC patients with advanced stage**

**Group (number of unfavorable genotype)** ^**a**^	**Death/total**	**HR (95% CI)** ^**b**^	***P*** **value**	**Recurrence/total**	**HR (95% CI)** ^**b**^	***P*** **value**
Group 1(0)	10/35	Ref.		11/35	Ref.	
Group 2(1)	15/39	1.66(0.73–3.78)	0.23	16/39	1.50(0.68–3.30)	0.31
Group 3(2)	84/169	*2.24(1.15–4.36)*	*0.02*	93/169	*2.33(1.24–4.38)*	*0.01*
*P* for trend			*0.01*			*0.003*

Significant *P* values (<0.05) are in italics.

*Abbreviations*: *CI*, confidence interval; *HR*, hazard ratio; *Ref.*, reference.

^a^Unfavorable genotypes: rs2304497 WW and rs9912300 WW.

^b^Adjusted by gender, age, hospital site, tumor position, TNM stage, tumor differentiation, and treatment after surgery where appropriate.

### Haplotype and diplotype of *ACLY* gene on overall survival and recurrence-free survival in CRC patients with advanced diseases

The haplotype and diplotype analyses were conducted to evaluate the combined effect of the two SNPs in *ACLY* gene on CRC overall survival. There were three haplotypes in order of rs2304497 and rs9912300 (TT, 82.1%; TG, 9.9%; and GG, 8.0%) and five diplotypes (TT-TT, 66.6%; TT-TG, 15.8%; TG-TG, 14.9%; TT-GG, 1.5%; TG-GG, 1.2%). Rare diplotypes (TT-GG, 1.5%; TG-GG, 1.2%) were removed from further analyses. As shown in Table 4, no significant combined effect was observed.

**Table 4:** **Haplotype and diplotype of ACLY gene and CRC survival**

**Group**	**Frequency (%)**	**Death**		**Recurrence**	
		**HR** ^**a**^ **(95% CI)**	***P*** **value**	**HR** ^**a**^ **(95% CI)**	***P*** **value**
Haplotype					
T-T	82.10	Ref.		Ref.	
T-G	9.90	0.92(0.66–1.29)	0.64	0.87(0.64–1.18)	0.36
G-G	8.00	0.86(0.59–1.26)	0.44	0.84(0.59–1.20)	0.34
Diplotype					
T_T-T_T	66.60	Ref.		Ref.	
T_T-T_G	15.80	0.90(0.61–1.33)	0.58	0.84(0.58–1.21)	0.34
T_G-T_G	14.90	0.80(0.53–1.22)	0.30	0.76(0.52–1.12)	0.16

Significant *P* values (<0.05) are in italics.

*Abbreviations*: *CI*, confidence interval; *HR*, hazard ratio; *Ref.*, reference.

^a^Adjusted by gender, age, hospital site, tumor position, TNM stage, tumor differentiation, and treatment after surgery where appropriate.

## Discussion

In the present study, we evaluated the effects of two functional SNPs in *ACLY* gene on the prognosis of a cohort of 697 Chinese CRC patients. We demonstrated that in CRC patients with advanced stage tumor (stage III + IV), the two SNPs (rs2304497 and rs9912300) were significantly associated with the OS and RFS. Furthermore, we identified an accumulative death risk and recurrence risk with increasing number of unfavorable genotypes. To the best of our knowledge, this is the first study to report that genetic variants in *ACLY* gene have a significant effect on the prognosis of patients with advanced stage CRC.

In most tissues, *de novo* fatty acid synthesis occurs at low rates since lipids are acquired via the circulation to support the needs of vegetative nonproliferating cells. In contrast, DNL occurs at very high rates in tumor tissues [23]. ACLY is in the first committed step of DNL pathway, contributing to the translocation of acetyl-CoA from the mitochondria to the cytosol. Recently, there has been growing interests in the impact of *ACLY* on cancer. Distinctive elevation of ACLY expression and activity has been reported in several types of cancer. Furthermore, it has been reported that *ACLY* inhibition with chemical inhibitors or siRNA can suppress tumor cell proliferation and induce apoptosis *in vitro* and *in vivo* [11,24]. Although the exact role of *ACLY* in tumorigenesis is yet unclear, previous studies have provided several plausible explanations, such as decreased glucose-dependent lipid synthesis [24] or fatty acid or cholesterol starvation [25], impaired glycolysis [26], inhibition of histone acetylation [27], mitochondrial ROS generation [28], and interception of PI3K/AKT signaling [29]. Our data from stratified analysis also suggest that adjuvant chemotherapy might not have obvious modifying effects on the prognostic significance of both SNPs. However, due to small number of patients in each subgroup, we have to interpret our stratified data with great caution. Further confirmation is warranted using larger patient population in future studies.

Recently, quite a few studies have demonstrated that SNPs in other DNL pathway genes are associated with the risk and prognosis of several tumors [30-33]. However, no studies have focused on the association between polymorphisms of *ACLY* genes and CRC prognosis until now. Our significant finding indicated that two SNPs in *ACLY* gene had a clear effect on OS and RFS of CRC patients with advanced stage tumor. As mentioned earlier, little is known to the association of *ACLY* gene with cancer prognosis. Report only indicates that *ACLY* expression correlated well with tumor grade, stage and poorer prognosis in non-small cell lung cancer [4]. Since SNPs play an important role in the variation of gene expression level and activity of proteins, it is reasonable to presume that SNPs in *ACLY* gene may affect the intracellular concentration of the metabolites through their impact on the enzyme activity. Variations of these metabolites would activate oncogenic signaling pathways at different levels in tumor cells, leading to different characteristics of tumor and distinctions in patient survival at last. Lin et al. have reported that triple arginine mutants of three lysine residues K540, K546, and K554 in ACLY protein result in an enhanced DNL and tumor growth both *in vitro* and *in vivo* [34]. rs2304497 in our study is a missense SNP that results in an amino-acid change of ACLY protein, which may lead to the alterations in structure and activity of this enzyme. Another SNP (rs9912300) in our study is located in the transcription-factor-binding site (TFBS), which may affect the transcription of *ACLY* and, ultimately, the level of protein expression. Different ACLY expression levels caused by gene polymorphisms may lead to the variation of metabolic pattern and other biological characteristics of CRC cells, thus resulting in different prognosis in patients. All these hypotheses need further experimental investigation in future study.

Our study has several strengths. First, the patient cohort in this study was relatively large and recruited from a single institution. The uniform standard operation procedures in cancer identification, pathological staging, and cancer treatment strategy make our findings more comprehensive and applicable to future clinical studies. Furthermore, the cumulative effects analysis established a novel combination of SNPs to predict the outcome of CRC patients with advanced stage tumor, which could be investigated further and included in a prognostic model to help clinicians in predicting outcomes for CRC patients with advanced stage tumor.

## Conclusions

Overall, as the first study observing the effect of *ACLY* gene polymorphisms on CRC prognosis in a Chinese population, our results strongly suggest that 2 SNPs of *ACLY* genes may be independent prognostic markers for recurrence and survival prediction in CRC patients with advanced stage tumor. These findings warrant further studies on the impact of these SNPs on the effectiveness of therapeutics against *ACLY* in CRC.

## Additional file

Additional file 1: Table S1. Associations between *ACLY* gene genotypes and clinical outcomes of CRC patients with/without chemotherapy.

## References

[1] Swinnen JV, Brusselmans K, Verhoeven G (2006). Increased lipogenesis in cancer cells: new players, novel targets. Curr Opin Clin Nutr Metab Care.

[2] Menendez JA, Lupu R (2007). Fatty acid synthase and the lipogenic phenotype in cancer pathogenesis. Nat Rev Cancer.

[3] Icard P, Poulain L, Lincet H (2012). Understanding the central role of citrate in the metabolism of cancer cells. Biochim Biophys Acta.

[4] Migita T, Narita T, Nomura K, Miyagi E, Inazuka F, Matsuura M (2008). ATP citrate lyase: activation and therapeutic implications in non-small cell lung cancer. Cancer Res.

[5] Wang Y, Shen L, Pang Y, Qiao Z, Liu P (2012). Prognostic and therapeutic implications of increased ATP citrate lyase expression in human epithelial ovarian cancer. Oncol Rep.

[6] Yancy HF, Mason JA, Peters S, Thompson CE, Littleton GK, Jett M (2007). Metastatic progression and gene expression between breast cancer cell lines from African American and Caucasian women. J Carcinog.

[7] Yahagi N, Shimano H, Hasegawa K, Ohashi K, Matsuzaka T, Najima Y (2005). Co-ordinate activation of lipogenic enzymes in hepatocellular carcinoma. Eur J Cancer.

[8] Varis A, Wolf M, Monni O, Vakkari ML, Kokkola A, Moskaluk C (2002). Targets of gene amplification and overexpression at 17q in gastric cancer. Cancer Res.

[9] Turyn J, Schlichtholz B, Dettlaff-Pokora A, Presler M, Goyke E, Matuszewski M (2003). Increased activity of glycerol 3-phosphate dehydrogenase and other lipogenic enzymes in human bladder cancer. Horm Metab Res.

[10] Halliday KR, Fenoglio-Preiser C, Sillerud LO (1988). Differentiation of human tumors from nonmalignant tissue by natural-abundance 13C NMR spectroscopy. Magn Reson Med.

[11] Hatzivassiliou G, Zhao F, Bauer DE (2005). ATP citrate lyase inhibition can suppress tumor cell growth. Cancer Cell.

[12] Siegel R, Desantis C, Jemal A (2014). Colorectal cancer statistics. CA Cancer J Clin.

[13] Center MM, Jemal A, Smith RA, Ward E (2009). Worldwide variations in colorectal cancer. CA Cancer J Clin.

[14] Dai Z, Zheng RS, Zou XN, Zhang SW, Zeng HM, Li N (2012). Analysis and prediction of colorectal cancer incidence trend in China. Zhonghua Yu Fang Yi Xue Za Zhi.

[15] Shin A, Jung KW, Won YJ (2013). Colorectal cancer mortality in Hong Kong of China, Japan, South Korea, and Singapore. World J Gastroenterol.

[16] Zaanan A, Dalban C, Emile JF, Blons H, Flejou JF, Goumard C (2014). ERCC1, XRCC1 and GSTP1 single nucleotide polymorphisms and survival of patients with colon cancer receiving oxaliplatin-based adjuvant chemotherapy. J Cancer.

[17] Tahara T, Okubo M, Shibata T, Kawamura T, Sumi K, Ishizuka T (2014). Association between common genetic variants in pre-microRNAs and prognosis of advanced gastric cancer treated with chemotherapy. Anticancer Res.

[18] Beuselinck B, Karadimou A, Lambrechts D, Claes B, Wolter P, Couchy G (2014). VEGFR1 single nucleotide polymorphisms associated with outcome in patients with metastatic renal cell carcinoma treated with sunitinib - a multicentric retrospective analysis. Acta Oncol.

[19] Yuan P, Wang S, Zhou F, Wan S, Yang Y, Huang X (2014). Functional polymorphisms in the NPAS2 gene are associated with overall survival in transcatheter arterial chemoembolization-treated hepatocellular carcinoma patients. Cancer Sci.

[20] Guo X, Li H, Fei F, Liu B, Li X, Yang H (2014). Genetic variations in SLC3A2/CD98 gene as prognosis predictors in non-small cell lung cancer. Mol Carcinog.

[21] Du X, Wan S, Chen Y, Qu P, Huang X, Yu X (2014). Genetic variants in genes of tricarboxylic acid cycle key enzymes predict postsurgical overall survival of patients with hepatocellular carcinoma. Ann Surg Oncol.

[22] Zhou F, He X, Liu H, Zhu Y, Jin T, Chen C (2012). Functional polymorphisms of circadian positive feedback regulation genes and clinical outcome of Chinese patients with resected colorectal cancer. Cancer.

[23] Kuhajda FP (2000). Fatty-acid synthase and human cancer: new perspectives on its role in tumor biology. Nutrition.

[24] Bauer DE, Hatzivassiliou G, Zhao F, Andreadis C, Thompson CB (2005). ATP citrate lyase is an important component of cell growth and transformation. Oncogene.

[25] Zaidi N, Royaux I, Swinnen JV, Smans K (2012). ATP citrate lyase knockdown induces growth arrest and apoptosis through different cell- and environment-dependent mechanisms. Mol Cancer Ther.

[26] Beckner ME, Fellows-Mayle W, Zhang Z, Agostino NR, Kant JA, Day BW (2010). Identification of ATP citrate lyase as a positive regulator of glycolytic function in glioblastomas. Int J Cancer.

[27] Wellen KE, Hatzivassiliou G, Sachdeva UM, Bui TV, Cross JR, Thompson CB (2009). ATP-citrate lyase links cellular metabolism to histone acetylation. Science.

[28] Migita T, Okabe S, Ikeda K, Igarashi S, Sugawara S, Tomida A (2013). Inhibition of ATP citrate lyase induces an anticancer effect via reactive oxygen species: AMPK as a predictive biomarker for therapeutic impact. Am J Pathol.

[29] Hanai J, Doro N, Sasaki AT, Kobayashi S, Cantley LC, Seth P (2012). Inhibition of lung cancer growth: ATP citrate lyase knockdown and statin treatment leads to dual blockade of mitogen-activated protein kinase (MAPK) and phosphatidylinositol-3-kinase (PI3K)/AKT pathways. J Cell Physiol.

[30] Eggert SL, Huyck KL, Somasundaram P, Kavalla R, Stewart EA, Lu AT (2012). Genome-wide linkage and association analyses implicate FASN in predisposition to uterine leiomyomata. Am J Hum Genet.

[31] Sinilnikova OM, McKay JD, Tavtigian SV, Canzian F, DeSilva D, Biessy C (2007). Haplotype-based analysis of common variation in the acetyl-coA carboxylase alpha gene and breast cancer risk: a case–control study nested within the European Prospective Investigation into Cancer and Nutrition. Cancer Epidemiol Biomarkers Prev.

[32] Sinilnikova OM, Ginolhac SM, Magnard C, Leone M, Anczukow O, Hughes D (2004). Acetyl-CoA carboxylase alpha gene and breast cancer susceptibility. Carcinogenesis.

[33] Nguyen PL, Ma J, Chavarro JE, Freedman ML, Lis R, Fedele G (2010). Fatty acid synthase polymorphisms, tumor expression, body mass index, prostate cancer risk, and survival. J Clin Oncol.

[34] Lin R, Tao R, Gao X, Li T, Zhou X, Guan KL (2013). Acetylation stabilizes ATP-citrate lyase to promote lipid biosynthesis and tumor growth. Mol Cell.

